# rLung, Mind, and Mental Health: The Notion of “Wind” in Tibetan Conceptions of Mind and Mental Illness

**DOI:** 10.1007/s10943-019-00775-0

**Published:** 2019-02-21

**Authors:** Susannah Deane

**Affiliations:** 1 Bath, UK; 2 Department of Religion and Theology, University of Bristol, Bristol, UK

**Keywords:** Medicine, *rLung*, Mental health, Mental illness, Mind, Psychiatry, Sowa Rigpa, Tibet

## Abstract

This article presents an analysis of the way in which *rlung* (“wind, breath”) functions as a mode of explanation for what Western medicine regards as “psychiatric” illness, based on field research on the topic of mental health, illness, and healing conducted within a Tibetan population in Darjeeling, northeast India. The article explores this notion of *rlung* and its relationship to body and mind, in order to examine its role in the causation and treatment of various forms of “mental illness”, before analysing some similarities and differences between *rlung*-related categories and biomedical classifications of mental illness.

English-language literature on Tibetan approaches to the mind and mental health often demonstrates a focus on some so-called spiritual factors, such as Tibetan Buddhist meditation practices or Buddhist perspectives on suffering and attachment, with titles such as Chögyam Trungpa’s *The sanity we are born with: A Buddhist approach to psychology*, or Dorjee et al.’s *The spiritual medicine of Tibet: Heal your spirit, heal yourself*.^1^ Here, they argue that “[t]he basic cause of illnesses according to Buddhist philosophy, psychology and medicine, is the ego, full of trivial pursuits and clinging attachments. It is this clinging nature that ultimately produces as confused mind and thereby all suffering and disease” (2005, p. 138). Indeed, the ultimate underlying causes of both physical and mental diseases are said to be the three “mental factors” (Tib. *nyon mongs*) of the Buddhist tradition: delusion (Tib. *gti mug*); attachment (Tib.*’dod chags*), and aversion (Tib. *zhe sdang*) (Epstein and Topgay 1982, p. 68).

I encountered some similar perspectives during my own research, examining the indigenous Tibetan medical tradition known as “Sowa Rigpa”,^2^ and understandings of mental health, illness, and healing within a Tibetan exile community in Darjeeling, northeast India in 2011 and 2012. Here, both religious and medical specialists as well as lay Tibetans described the importance of factors such as keeping the mind “calm” and remaining detached from material things in order to maintain health—mental health particularly. However, there is another factor involved here too: the Tibetan notion of “wind” (Tib. *rlung*), which is fundamental to Tibetan conceptions of the mind and body, and is thus integral to understandings of mind, consciousness, and mental health. One of three “energies” (Tib. *nyes pa*) in the body, wind is described in detail in the predominant medical text, the *rGyud bZhi* (*Four Tantras*), where there are descriptions of sixty-three kinds of disorder related to its disturbance (Millard 2007). Indeed, in narratives about mental illness (Tib. *sems nad*) in the Tibetan context, it is this concept which often recurs, as both lay Tibetans and medical specialists discuss prevention, causation, and treatment. Here, the single term “*rlung*” is often used to denote both the concept of “wind” itself, and also any condition/illness understood to result from a disturbance to it. Thus people will describe “having wind” or perhaps “high wind” to denote a wind-related condition.

In this article, I will use examples from my fieldwork within a Tibetan community in Darjeeling to illustrate some contemporary understandings of this notion of “wind”. In this high-altitude corner of West Bengal, a significant community of first-, second-, and third-generation exiles live both in and around the town itself, and at the nearby Tibetan Refugee Self Help Centre. In Darjeeling, I interviewed medical and religious professionals as well as lay members of the community on the topic of mental health, illness, and healing, and the notion of “wind” was widely discussed in this context. However, Darjeeling is an area of cultural and medical pluralism, where the Tibetan community lives amongst the majority Nepali and Indian population. As a result, the town is home to an array of medical and healing facilities, including two Tibetan Sowa Rigpa clinics—a branch clinic of the Tibetan government-in-exile-affiliated Men-Tsee-Khang (MTK), and a clinic run by the independent Chagpori Tibetan Medical Institute (CTMI)—and a plethora of Ayurvedic and biomedical clinics and hospitals (both private and government). Tibetans in this area are therefore exposed to a variety of medical systems, each with its own explanatory frameworks regarding health and illness. What was notable during my fieldwork was the endurance of long-standing Tibetan notions of mind and body, particularly in relation to this notion of wind*. rLung* is understood to be intimately connected to the mind, and we often hear the explanation that “the mind rides the wind like a man rides a horse”: in other words, the man (mind) needs the horse (wind) for movement/energy, but the horse (wind) also needs the man (mind) for direction.

It is important to note here that Tibetan understandings of “wind” have been adapted from Indian Tantric and Ayurvedic ideas about the mind and body. As a result, the Tibetan notion of “*rlung*” is in fact a conflation of two different Indian/Sanskrit concepts: *vāta* and *prāṇa*. *Vāta* refers to one of the three Ayurvedic *doṣa* which correspond to the three Tibetan *nyes pa* of wind, bile, and phlegm in the medical sense. *Prāṇa* is a Tantric concept often translated as “life force” or “breath”, and it is the focus of many Tantric meditative and bodily practices which aim to manipulate its movement within the body. There are many such Tantric practices in the Tibetan Buddhist tradition too, but in this article I will give an overview of the notion of *rlung* in the medical context, examining how this conception of “wind” relates to the mind and consciousness, and exploring what this means in terms of mental health and illness. I will give an overview of the different types of wind said to flow in the body and the diverse physical and psychological symptoms understood to result from its disturbance, before exploring the diagnosis and treatment of wind-related mental illness in the Sowa Rigpa medical tradition. Finally, I will address the question of whether it is in fact possible to equate some of these *rlung*-related illnesses with biomedical categories of mental illness, as has been suggested by a number of Tibetan and Western practitioners and researchers.

## *rLung* and “Humoral” Theory

In the Sowa Rigpa tradition,^3^ as in the Ayurvedic system, “wind” is one of three “energies” (Tib. *nyes pa*) of wind (Tib. *rlung*), bile (Tib. *mkhris pa*), and phlegm (Tib. *bad kan*)^4^ described in the *Four Tantras*. This twelfth-century, four-volume medical text encompasses original Tibetan medical concepts alongside influences from Sanskrit and Chinese texts (Emmerick 1977; Ga 2010; see also Ga 2014; Gerke 2014; Gyatso’s recent 2015 work on the development of medical theory in Tibet in the twelfth to eighteenth centuries). The text describes the diagnosis and treatment of illnesses encompassing both physical and psychological symptoms and is studied as part of Sowa Rigpa practitioners’ training. Any of these three energies can be a potential cause of disease, in a system where all diseases are said to be linked—to at least some degree—to disturbance in one or more of these *nyes pa* (Epstein and Topgay 1982, p. 69). Indeed, medical practitioners and lay Tibetans alike will often describe ill-health in terms of a “disturbance” in wind, bile, or phlegm—explanations I heard numerous times over the course of discussions on health and illness in Darjeeling. Whilst anyone can be subject to disturbance in any one or more of these *nyes pa* of wind, bile, and phlegm, the *nyes pa* need not be the sole cause of an illness, but may be working alongside some other causative or contributory factor(s)—such as poor diet, karma, or spirit affliction for example—to cause illness.

Wind, bile, and phlegm are related to different aspects and functions of the mind and body: Dorjee et al. describe them as “very subtle, pervasive energies, currents or forces which are vital to the body and which operate not only on the physical level, but also on the psychological and spiritual levels” (2005, p. 148). For example, wind is said to be responsible for the inhalation and exhalation of the breath and the physical movement of the body. Bile is responsible for the digestive system, thirst, and hunger, and for developing physical heat in the body, and is understood to underpin bravery and intelligence. Phlegm is responsible for maintaining the stability of the body and mind, inducing sleep and lubricating the joints (Dorjee et al. 2005, pp. 149–150). In addition, these three *nyes pa* are associated with different periods in a person’s lifetime, with different times of day and the different seasons. For example, wind is associated with older age/the latter portion of life, dawn and dusk, and the rainy season—meaning that individuals are more likely to suffer from a wind disturbance at a wet time of year, and in a rainy, windy place or during the monsoon season (Dorjee et al. 2005, pp. 273–274); and also that older people are more prone to wind disturbances than younger people.

The Tibetan term *nyes pa* is a direct translation of the Sanskrit *doṣa*, and it has traditionally been translated into English as “humour”—by both Tibetan and non-Tibetan practitioners and scholars. However, this translation is somewhat problematic, and it has been suggested that this comparison with Western humoral theory (such as that found in Greek/Hellenic medicine) is in fact rather misleading (Ozawa-de Silva and Ozawa-de Silva 2011, p. 104; see also Gyatso 2005, pp. 109–110). Indeed, as Samuel has pointed out, the word *nyes pa* actually translates literally into the English terms “fault” or “weakness” (2001, p. 255), and it has more recently been translated as “defective energies” by some Tibetan doctors (Kloos 2010, p. 17 [note 20]). Furthermore, we find that much of the discussion on *nyes pa* in the English-language literature on the topic focuses on the notion of humoral “balance” versus “imbalance”. For example, the late Sowa Rigpa practitioner Pema Dorjee tells us that, “In a healthy body the balance between the three *nyespas* is maintained and appropriate qualities of each are present. This balance, however, is a precarious one, and the increase, decrease or erratic movement… moves the body towards a diseased condition” (Dorjee et al. 2005, p. 148). In fact, this interpretation about “balance” and “imbalance” is *also* rather problematic—Samuel argues that in contrast to the Galenic and Islamic humoral systems (in which the humours can indeed be “balanced” or “unbalanced”) in the Tibetan system, these three constituents are often understood to actually be “exclusively negative in nature” (2001, p. 255). Thus whilst this language of “balance” remains fairly common in discourses from both Tibetan and non-Tibetan doctors and researchers, a more accurate way to describe this situation is perhaps through notions of “excess”, “deficiency” or “disturbance” in one or more of the *nyes pa* (Yoeli-Tlalim 2010, p. 320).

Within this tripartite system of wind, bile, and phlegm, it is the wind “current”, and the “channels” (Tib. *rtsa*) in which the wind is said to flow, which are the focus of much of the discussion—particularly when we are talking about the mind, consciousness, and mental health. This is because, as I mentioned above, the wind is said to be intimately related to the consciousness, with the mind “inseparably linked” to the body through the wind (Rabgay 1984, p. 50). In addition, wind itself is a concept which spans Tibetan medical and religious notions of the mind and body—in part as a result of its dual origins in Indian Tantra and medicine. As Epstein and Topgay explain,

Both medical and religious texts lay a strong emphasis on these currents of psychic energy…. Tibetan medical texts are filled with descriptions of the manifestations of dysfunction of the pranic [wind] flow, and Tantric religious texts voluminously elucidate the reorganization of these currents that occur in death or in meditation. An understanding of the Tibetan approach to mind is not possible without an appreciation of the character of these pranic [wind] currents. (1982, p. 68).

Thus we find Epstein and Topgay describing the system of wind channels as a kind of “psychic nervous system” (1982, p. 68), and it is understood that we can manipulate the consciousness through manipulation of this wind. In this context, we find practices aimed at directing the wind currents in the body in the spheres of both health and religious practice—conducted both “for the purpose of health maintenance”, but also ultimately, for “spiritual liberation” (Yoeli-Tlalim 2010, p. 320).

As I mentioned above, *rlung* is understood to flow through “channels” (Tib. *rtsa*) in the body. These channels are said to number in the tens (or even hundreds) of thousands (see, for example, Wangyal and Dahlby 2002, p. 81), and are often described as “undetectable” and “immeasurable”. Three of these channels are particularly implicated in mental health and illness: the central channel (Tib. *rtsa dbu ma*) and the channels each side of this—one each to the left (Tib. *rtsa rkyang ma*) and the right (Tib. *rtsa ro ma*) (Drungtso 2004, pp. 90–91). These left and right channels intersect with the central channel at a series of confluence points, commonly referred to as “chakras” (Tib.*’khor lo*, Skt. *cakra*, energy centres), but again there is debate over exactly how many of these chakras exist. Over the last few decades, as many Sowa Rigpa practitioners have become increasingly knowledgeable about Western scientific theories of the body, health, and illness, and many Westerners have become interested in Sowa Rigpa (and Tibetan Buddhist Tantra), there have been a number of attempts to “map” these channels onto Western anatomical maps and biomedical^5^ understandings of the body—by Sowa Rigpa practitioners and also by researchers. For example, some contemporary Tibetan authors explain the channels as part of the nervous system (Dorjee et al. 2005, p. 164) or the blood vessels, nerves and lymphatic system of biomedical anatomy (Wangyal and Dahlby 2002, p. 81). However, it remains difficult to find direct correlations between the Tibetan and biomedical systems, especially when we start to explore the “characteristics” of these wind currents as they are described in the *rGyud bZhi* medical text. For example, “wind” is said to have six characteristics—rough, light, cold, subtle, hard, and mobile (Drungtso 2004, p. 99)—and there are in fact five principal wind currents described in the *Four Tantras*, which derive from the five *prāna* in the Indian system: ascending (Tib. *gyen rgyu rlung*); pervasive (Tib. *khyab byed rlung*); fire-accompanying (Tib. *me mnyam rlung*), descending (Tib. *thur sel rlung*) and life-sustaining (Tib. *srog (’dzin) rlung*). Each of these wind “currents” is understood to be located in a different area of the body, associated with a different element, and related to different functions of the body and mind. As a result, disruption or disturbance in any one (or more) of the wind currents will result in diverse physical and psychological symptoms. These five wind currents have been described in detail elsewhere (see, for example, Yoeli-Tlalim 2010), so here I will simply give an overview of the five currents, before focusing on the “life-sustaining wind”, which is at the forefront of many explanations of mental health and illness.

### Ascending Wind (Tib. *Gyen rgyu rLung*)

Localised in the chest, this wind current also circulates in the region of the throat, nose, and tongue (Epstein and Topgay 1982, p. 72) and is associated with the element of fire (Drungtso 2004, p. 99). The ascending wind produces the voice, clears the sensory organs and the mind, assists memory and aids willpower. Disruption to this wind can be caused by factors including the forcible withholding of burping or vomiting, “crying or laughing loudly”, or lifting or carrying a load too heavy for the individual (Dorjee et al. 2005, 168). Symptoms of disruption in the ascending wind include stammering or difficulty speaking, loss of physical strength, facial paralysis, and memory loss (Dorjee et al. 2005, p. 168).

### Pervasive Wind (Tib. *Khyab byed rLung*)

Localised in the region of the heart, the pervasive wind pervades the whole body and is associated with the element of space (Drungtso 2004, p. 100). This wind current facilitates movement in the extremities (for example, through walking, stretching, lifting, bending and the opening and closing of the eyes and lips), and is responsible for the circulation of the blood and lymph in the body (Dorjee et al. 2005, p. 169). Disruption can be caused to the pervasive wind by factors including poor food, overwalking, sitting too long on wet ground, excessive physical work or exercise, or psychological factors such as sudden fear (Dorjee et al. 2005, p. 169) or “shock, fright, depression, or phobic behaviour” (Epstein and Topgay 1982, p. 75). Symptoms of such disruption can include discomfort in the heart, unconsciousness or fainting, meaningless talking, and restlessness (Dorjee et al. 2005, p. 169), “decreased mental functioning”, “unsubstantiated fears” (1982, p. 75), and panic attacks, (Yoeli-Tlalim 2010, p. 320).

### Fire-Accompanying/Metabolic Wind (Tib. *Me mnyam rLung*)

Fire-accompanying wind is localised around the navel and stomach and is associated with the air element (Drungtso 2004, p. 100). This wind current supports digestion and is responsible for bowel movement (Dorjee et al. 2005, p. 169; Drungtso 2004, p. 100). Disruption to its functioning can be caused by factors including the intake of heavy and/or stale foods or sleeping during the daytime (Dorjee et al. 2005, p. 169), and symptoms of this disruption may include poor digestion and/or a loss of appetite or vomiting (Dorjee et al. 2005, p. 169).

### Descending Wind (Tib. *Thur sel rLung*)

Residing in the colon, bladder and reproductive organs (Yoeli-Tlalim 2010, p. 320), the descending wind is associated with the earth element (Drungtso 2004, p. 100). This wind current facilitates the expulsion of urine and faeces, is responsible for menstruation, and helps in childbirth (Dorjee et al. 2005, p. 170). Disruption here can be caused by factors including the forcible withholding of urine, faeces, or semen. Symptoms of such disturbance include aching in the hips, constipation, and gas accumulation in the stomach, as well as more psychological symptoms such as restlessness and fear (Dorjee et al. 2005, p. 170), “psychological and emotional distress, such as jealousy, fear and worry” (Yoeli-Tlalim 2010, p. 320).

### Life-Holding/Life-Sustaining Wind (Tib. *Srog’dzin rLung*)

Finally, we have the “life-holding” or “life-sustaining” wind (Tib. *srog (’dzin) rlung*). Described variously as being located in the centre of the body (Yoeli-Tlalim 2010, p. 320) or the heart,^6^ or at the crown of the head, and associated with the element of water (Drungtso 2004, p. 99), this is the wind current most often implicated in mental health and illness. Thus even though, as we can see from the descriptions above, psychological factors can be both cause and symptom of disturbance in several different wind currents, when discussing mental health and illness with both lay Tibetans and Sowa Rigpa practitioners, it is the life-sustaining wind current which is often mentioned—implicated in conditions both mild and severe. Indeed, Epstein and Topgay tell us that,

Tibetans conceive of most anxieties and depressions in terms of a disturbance in sok-*r*lung… When the life-bearing current is more seriously affected, i.e., through invasion of its channels by other increased currents, symptoms of violent or hysterical behavior may appear. If severely affected, psychosis may result (1982, p. 74).

The life-holding wind flows in the “life-holding channel” (Tib. *srog tsa*) which is said to run from the throat down to the chest, and is understood to support the life force and hold the consciousness. Yoeli-Tlalim describes how this wind current “governs the body/mind system” (2010, p. 320) and, because this current is so closely linked to the consciousness, perhaps unsurprisingly, any disturbance in it can lead to problems such as confusion, auditory and/or visual hallucinations (Yoeli-Tlalim 2010, p. 320), restlessness, stress, anxiety, depression, dizziness, and insanity—as well as physical symptoms such as difficulty in drawing breath or swallowing food or drinks (Dorjee et al. 2005, pp. 167–168), reduced appetite, fatigue, and headache (Jacobson 2007, pp. 235–237). Factors understood to disrupt the flow of this wind current include eating poor quality, malnutritious food or fasting for long periods (Dorjee et al. 2005, p. 168).

## *rLung*-Related Illness in the Sowa Rigpa Tradition

In addition to the quite specific factors listed above as potential causes of disturbance in the various *rlung* currents, what we might call “mental stresses” of various kinds (for example, tension, worry or low mood) are understood to potentially disturb the flow of *any* of the various wind currents. It is clear from these descriptions that both physical and psychological factors may negatively impact the flow of the winds in the body. However, there is often a focus on the *psychological* factors when we talk about *rlung* and conditions related to its disturbance—much more so than in discussions on bile or phlegm.

In Darjeeling, I discussed *rlung*-related illness with Tibetan informants, who were often quite familiar with both its various causative factors and its treatment. For example, when I discussed this topic with Dolkar (55),^7^ a second-generation Tibetan exile living at the TRSHC, she gave a fairly common example, describing a cause of *rlung* disturbance: “If I am worrying about my son in Delhi [in the past he had been very ill with TB]—if I am worrying about his health, and what will happen to him, I will get *rlung*… [But] if you can control [your worry, your mind], then it’s ok”. Similarly, at the CTMI clinic, Sowa Rigpa practitioner Amchi^8^ Teinlay Trogawa explained to me that “a very big shock” or a “burden on the mind” over a long period of time could cause a disturbance to one or more of the *rlung* currents: “When the mind is disturbed or worried—due to family worry, or work stress, or whatever it is—that would disturb the energy of the wind, and then illness would manifest”.

In addition, as we saw from the lists of symptoms associated with disturbance in the various wind currents described above, a disturbance in the wind is also quite likely to *cause* psychological symptoms of various kinds. Dorjee et al. provide an explanation of how this can occur: “An increase in this [*rlung*] energy can cause us to become unstable and susceptible to external occurrences… The mind will weaken and we find that we are increasingly vulnerable to sadness and depression” (2005, p. 139). Thus we see a kind of circular system, whereby various mental or emotional states can disturb the wind, and disturbed wind can lead to psychological disturbance. As Epstein and Topgay explain, “mental disturbance is said to be reflected in an alteration in the flow of *r*lung, and likewise, disturbance of *r*lung anywhere within the organism is said to produce correlative mental or emotional disturbances” (1982, p. 72). Indeed, a healthy, functioning mind is said to depend on the undisturbed flow of the various wind currents (Epstein and Topgay 1982, p. 71), and thus any *disturbance* in the wind currents—and particularly the life-holding wind current—has clear implications for mental health. Interviewing Amchi Sonam Dolma^9^ in Dharamsala in 2012 she told me: “Of course there’s no denying that the principal cause of all mental problems is *rlung*… *rLung* is the main factor which disturbs the person’s sanity”. Thus we see that whilst it may be true, as I noted above, that many illnesses are said to be ultimately caused by the mind, as we can see, this is often mediated by the *rlung*: disturbed by emotions and thoughts, the disordered *rlung* may cause (or contribute to) illness. In addition, wind, bile, and phlegm are said to be ultimately caused by the three “mental factors” of delusion, attachment, and aversion, with the fundamental role of these mental factors in the formation of *rlung* described in the *rGyud bZhi* text (Ga 2010, p. 182). Thus we have a situation where, as Clifford summarised: “[e]motional imbalance produces humoral imbalance that is manifested as a psychiatric disturbance” (1989, p. 139).

Dorjee et al. have compared disordered wind energy to the energy of a hurricane, “which twists and uproots trees, and collapses buildings… a beautiful flower destroyed by violent winds is the perfect image of the damage caused by a *rLung* disordered state of mind” (2005, p. 163). Thus for example, when wind has the characteristic of being “mobile”, it is continuously fluctuating, and the person may consequently suffer from “delirium, hallucination and hysteria”. Such a patient, they tell us, is “typically talkative and always on the move” (Dorjee et al. 2005, p. 151). Similarly, in Darjeeling, Amchi Teinlay Trogawa explained that when *rlung* is mobile, it is not stable—“therefore [the patient] forgets things”, and “sometimes [the patient is] aggressive, [and/or] talking to themselves”.

This concept of wind and the symptoms and conditions related to its disturbance are generally well-understood by lay Tibetans as well as Sowa Rigpa practitioners. Yoeli-Tlalim describes, for example, the fairly common self-diagnosis by lay Tibetans of “high wind” to suggest emotional distress (2010, p. 321), with explanations such as “I have high wind today” indicating a period of worry or stress. This was certainly something I came across during my own research too, where lay and professional Tibetan informants alike used such explanations to describe or explain their state of mind. Working in Darjeeling, I was exploring ideas about “mental illness”^10^ quite broadly, but as I discussed this topic with lay Tibetans as well as medical and religious specialists, it was “wind” and wind disorders which came up most frequently.

I have described above some of the ways in which a disturbance in the wind can manifest in both physical and psychological symptoms, and this is indicative of the Sowa Rigpa approach, which does not separate out mental and physical illnesses into different sections within the medical text. In Darjeeling, Dawa, a 51-year old monk, explained to me, “*Sems nad* [mental illness] is very interesting—body and mind are inseparable: one affects the other; one is dependent on the other”. Thus a wind condition (similarly to a bile- or phlegm-related condition) may be characterised by both physical and psychological symptoms, as we saw above. One of the ways in which these diverse symptom clusters are explained is that when the wind is “erratic”, it “can send wrong information to the sense organs” (Dorjee et al. 2005, pp. 163–164)—as Dorjee et al. explain, this “erratic” nature of *rlung* “can thus result in double vision, seeing ghosts or hearing abnormal sounds—when the *rlung* is disturbed, they explain, it “plays tricks with the mind”. Memory, which is supported by the heart, may be impaired, and the disordered *rLung* can also produce fearful dreams” (Dorjee et al. 2005, pp. 163–164).^11^

## Diagnosis and Treatment of Wind Disturbances in the Sowa Rigpa Tradition

Diagnosis in the Sowa Rigpa tradition is predominantly conducted via the reading of the pulse and/or urine (and sometimes also the tongue) alongside questioning of the patient in order to determine the patient’s constitution and the nature of their condition. As with other types of illness described in the *rGyud bZhi*, treatment for wind disorders tends to favour a four-pronged approach, encompassing diet, medicine, behaviour, and physical therapy (Epstein and Topgay 1982, p. 76). However, depending on the case, *rlung* disorders may be hard to treat. At the CTMI clinic in Darjeeling, Amchi Lobsang Samten explained that whilst some cases of *rlung* are easily treated, others may take months—or even years—to treat effectively. At the MTK clinic, Amchi Lobsang Thubten explained that, depending on the case, a combination of “external [i.e. physical] therapies” and “counselling”^12^ were sometimes needed alongside any herbal medicines to successfully treat a *rlung* condition. “External therapies” for *rlung*-related conditions include the application of oily, hot poultices to the *rlung* points (for example, at the sixth and seventh cervical vertebrae) (Dorjee et al. 2005, p. 265), massage, moxibustion or needle therapy (Epstein and Topgay 1982, p. 76). Prescribed herbal medicines, of which there are many described in the text, should have sweet, sour, salty taste, and have an oily, heavy, and/or smooth quality (Dorjee et al. 2005, p. 262), and suitable medical compounds traditionally include agarwood (Tib. *a gar*), nutmeg, cloves, asafoetida and/or molasses (Dorjee et al. 2005, p. 262).

According to this four-part approach, there is also much that the patient him/herself can do to improve his/her condition. For example, a suitable diet for patients with *rlung* disturbance should be high in protein and include what are understood to be “nutritious” foods—for example “oil, butter, mutton with vegetables, soups, porridge, hot milk and a little alcohol” (Dorjee et al. 2005, p. 261). In terms of behaviour, patients are advised to “spend time with close, loving friends in a warm, calm and quiet place… kept free from stress and tension, entertained with music, interesting stories and amicable conversation” (Dorjee et al. 2005, p. 260). Sufferers should have a comfortable bed to induce undisturbed sleep, and a warm environment which is neither too light or too bright (Epstein and Topgay 1982, p. 76). Moreover, with wind conditions understood to be caused, at least to some extent, by certain psychological states, emotions or mental activities (such as worry), there is often reference to psychological factors in its treatment too. Certainly in Darjeeling, both practitioners and lay Tibetan informants highlighted the importance of emotional support for people suffering from such conditions, with several individuals describing the importance of helping afflicted individuals to avoid dwelling on problems or upsets, and encouraging patients to keep their mind “calm”. Indeed, as I noted in the introduction, there is often a focus on the role of the mind here, and Dorjee et al. suggest that “it is the mind that is the key to healing our worries” (2005, p. 139)—wind disturbances, he tells us, can be “controlled by both medicine and meditation” (2005, p. 167).

## Biomedicine and “Wind”

As I mentioned above, a number of Western and Tibetan Sowa Rigpa practitioners and researchers have made attempts to “map” the *rlung* and its channels onto biomedical notions of anatomy. Similarly, there has been a fair bit of discussion on whether it is possible to compare the various *rlung* diagnoses with biomedical categories of mental illness from the World Health Organization’s *International classification of Disease* (ICD)^13^ and/or the American Psychiatric Association’s *Diagnostic and statistical manual of Disease* (DSM)^14^ classification systems. In the culturally and medically pluralistic context of Darjeeling, many patients consulted both biomedical and Sowa Rigpa practitioners (sometimes also alongside other medical and/or religious practitioners), therefore receiving diagnoses from both systems. Indeed, as in many cultural contexts, where there are multiple medical (and religious) systems operating, this is not unusual, and in Darjeeling, I was interested to explore the different perspectives on the similarities and differences between these Tibetan and Western approaches to mental illness.

Interviewing both lay Tibetans and Sowa Rigpa practitioners in and around Darjeeling, I found many people quite familiar with Western notions of “stress”, “anxiety” and “depression” (and sometimes also “psychosis”)—particularly those with a good knowledge of English or a significant amount of contact with Westerners. Indeed, a number of interviewees drew comparisons between wind disorders and the broad biomedical categories of “anxiety” or “depression”, sometimes also using the Tibetan and English terms interchangeably. For example, when I interviewed Metok (63), a former nurse who had received her training in the UK many years before, she suggested that *srog (’dzin) rlung* disorder is “maybe like depression”. And, when I discussed mental illness with Tibetans in Darjeeling, it was *srog (’dzin) rlung*—“life-holding wind disorder”—which was discussed perhaps the most frequently. When I interviewed Dolma (31), a second-generation Tibetan exile fluent in English, she referred to *srog rlung* in English as “anxiety disease” and the combination English-Tibetan “anxiety *na tsha*” (literally, “anxiety illness”). Dolma explained that this condition is usually caused by “loneliness”, “too much thinking”, and/or what she referred to as a “competitive mind”. She told me that a friend of hers, Dawa (aged approximately 30), suffered from this, describing how he frequently felt “panic”—“especially in a crowded place, or when he goes in a car or jeep”, with symptoms that come and go. Whilst Dawa was under the care of a biomedical doctor, Dolma explained that in fact he did not take any (biomedical or other) medicine for his condition, as his doctor had advised him that it was better to manage without medicine if possible—indeed, she explained that Dawa himself felt that the best treatment for such conditions was “meditation”.

Dolma is not alone in making this equivalence between *srog rlung* and anxiety—indeed, a number of Tibetan and non-Tibetan Sowa Rigpa practitioners and researchers have drawn similarities between this diagnosis and biomedical categories of both anxiety and depression. It is clear from the above descriptions of *srog rlung* and the consequences of its disturbance that some of the symptom clusters certainly suggest such similarities. Discussing *srog rlung* with Amchi Teinlay Trogawa, director of the CTMI and a practitioner at its clinic in Darjeeling, he described symptoms including: “high and low mood”, “disturbed sleep”, and a “blocked feeling in the chest region”, also suggesting that for some sufferers, perhaps “in a closed room…. they think that they are getting suffocated”. He described physical symptoms too, for example, the “*rlung* points”—points on the body specifically related to the *rlung* currents—will likely be “very sensitive and tender”. At the Darjeeling MTK clinic, Amchi Lobsang Thubten explained that a patient with *srog rlung* will likely display a range of symptoms, such as sadness, restlessness, lack of appetite, inappropriate emotions or unusual aggression. He described how sufferers may also have a fear of being alone or in a crowd and may struggle with concentration and/or memory, perhaps also talking to themselves. Some sufferers, he explained, may even be suicidal. Physical symptoms described by Amchi Lobsang Thubten included heavy breathing, and a desire to stretch and/or yawn.

As we can see then, some of these symptoms show considerable similarity with the symptomatology of the various categories of depression and anxiety which we find in the biomedical ICD and DSM classification systems.^15^ Indeed, following research in Darjeeling in the 1990s, Jacobson described a “significant overlap” between Tibetan categories of *rlung* illness and “biomedical constructions of depression, anxiety and somatization”, suggesting that *srog rlung* is “most similar” to “major depressive and anxiety disorders” (2000, p. 529). However, there are a couple of issues here, as we can see from these lists of symptoms above. Firstly, as I have already mentioned, it is notable that in the Sowa Rigpa system—in contrast to the biomedical system—there is no demarcation into “physical” and “mental” illnesses—instead, conditions such as a *srog rlung* disturbance are understood to encompass a combination of both physical and psychological symptoms, as we saw above. Secondly, it seems that the boundaries of these Tibetan and biomedical illness categories are often drawn in different places. For example, as Epstein and Topgay describe above, disturbance to the *srog rlung* can lead to “common anxieties” and “minor depressions”, as well as “psychosis”, depending on the severity of the disturbance (1982, p. 74), and some Sowa Rigpa practitioners have described some such cases of *srog rlung* as “schizophrenia” (Rosenbush 2013). Observing a practitioner working in a Sowa Rigpa clinic in Edinburgh (UK), Millard found most mental disorders that patients presented with were diagnosed as being related to wind—particularly the *srog rlung*. However, he observed that life-holding wind disorders can take “various forms from mild depression and anxiety to extreme mental disturbance” (2007, p. 258), and Iin India, both Amchi Teinlay Trogawa (who had a very good knowledge of English and experience of treating foreign patients in the West) and Amchi Sonam Dolma named “depression” and “schizophrenia” as different kinds of *srog rlung*.

Similarly, biomedical diagnostic categories also do not necessarily fit easily into the Sowa Rigpa categorisation system. For example, Epstein and Topgay have suggested that “a fully developed case of clinical depression” (from a biomedical perspective) “could represent imbalance of at least four of the five major pranic [*rlung*] currents”, where for example, a disturbance in the *srog rlung* results in a “depressive mood state”, and disturbance in the fire-accompanying/metabolic wind results in a loss of appetite (1982, p. 75). The key difference, of course, is that in contrast to the biomedical classification system, where “minor depression” and “psychosis” are viewed as very different illnesses, and categorised in different sections of the texts,^16^ the Sowa Rigpa category of *srog rlung* is on a *continuum*. Here, a mild disturbance in the *srog rlung* may lead to symptoms such as sadness, depression, and/or anxiety, but a more severe disturbance might lead to far more severe symptoms—some of which biomedicine classifies as defining “psychotic” states (for example, hallucinations or delusions). Thus we have one Tibetan category spanning several biomedical ones. Millard concludes that while “it would be difficult to find Tibetan categories of mental illness which correspond exactly with the categories listed in the psychiatric section of the ICD10”, we *can* draw comparisons between “the Tibetan nosological category of *srog rlung*” and symptoms related to certain of its biomedically classified mental illnesses—particularly those in sections F2 (schizophrenia, schizotypal, and delusional disorders), F3 (mood (affective) disorders), and F4 (neurotic, stress-related, and somatoform disorders) (2007, p. 279)—a wide range of conditions indeed.^17^

## Conclusion

What I hope has become clear from the discussion here is just how fundamental this notion of *rlung* is to any exploration of Tibetan conceptions of the mind and consciousness—and therefore how integral *rlung* is in Sowa Rigpa approaches to mental health and illness. This “energy” (or “fault”) is only one of the many factors which can be involved in health and illness (mental or physical), but it is the one which is at the forefront of many discussions about *mental* illness—amongst both Sowa Rigpa practitioners and lay Tibetans. With a number of different wind currents understood to reside in the body, located in different places and related to different functions of the mind and body, lay Tibetans will not necessarily have an in-depth understanding of these. However, all of the Tibetans I interviewed in Darjeeling had at least a general knowledge of the principles of this system, and it is this concept which forms much of Tibetan medicine practitioners’ and lay Tibetans’ explanations of mind and mental health.

What should be clear is that *rlung* is a concept which brings together physical and psychological aspects of the body. As such, it is perhaps not surprising that both physical and psychological factors can lead to a disturbance in its flow, and also that resulting symptoms may be physical and/or psychological, ranging from poor digestion or vomiting to “depression” or “insanity”. As a result, treatment usually takes a several-pronged approach, frequently encompassing herbal medicine, physical therapy, dietary advice, and/or suggested activities to support the person emotionally. Thus the notion of the mind as the root of all (mental and physical) illness which I described at the beginning of this article can be understood in several ways. Not only from a Tibetan Buddhist perspective can we view the mind as the root of all suffering—as Dorjee et al. state, the “clinging nature that ultimately produces as confused mind and thereby all suffering and disease” (2005, 138)—but from an understanding of anatomy in Sowa Rigpa, we can see how the mind and body are intimately linked through the medium of *rlung.* Thus where the mind (or body) is disturbed in some way, the wind will become disturbed as a result, and this wind disturbance can lead to a multitude of psychological and physical symptoms.

Whether or not we can equate some of the diverse *rlung* conditions described above with any biomedical categories of mental illness remains an open question. With the underlying conceptions of anatomy significantly different in these two systems, and the boundaries of these categories drawn in often very different places, it remains rather problematic. However, with clear overlaps between symptomatology of some Sowa Rigpa conditions and some biomedical categories such as depression and anxiety, we might want to draw some comparisons. Moreover, a deeper examination of Tantric notions of *rlung* can perhaps shed further light on the possible relationships between the system of *rlung* and some particular Western concepts of anatomy, as Geoffrey Samuel explores in his article in this volume.

## Footnotes

1 Also notable is Terry Clifford’s *Tibetan Buddhist medicine and psychiatry: The diamond healing* (1989), one of the first English-language books by a Western author on the topic of mental illness in the Tibetan tradition.

2 Tib. *gSo ba Rig pa*, “knowledge/science of healing”.

3 This medical tradition is practised across the Himalayan region, from Ladakh in the West, across the Tibetan plateau to Bhutan in the East and Mongolia in the North. Whilst there is considerable variation in its practice in different regions, what is common is its use of the *rGyud bZhi* text, parts of which are usually memorised by medical students.

4 It is worth noting that these translations into “wind”, “bile”, and “phlegm” are not without problem (Yoeli-Tlalim 2010, p. 319). However, as these English terms are the most common translations of the Tibetan concepts of *rlung*, *mkhris pa*, and *bad kan*—used by both Tibetan and non-Tibetan specialists—I shall follow this convention here.

5 I use the terms “biomedicine” and “biomedical” here to refer to the system of medicine based on the application of the principles of the natural sciences, especially biology and biochemistry (See Merriam-Webster’s online dictionary definition: http://www.merriam-webster.com/medlineplus/biomedicine).

6 Notable here is the fact that in the Tibetan perspective, the seat of the mind (*sems*) is the heart (not the brain).

7 The names of all informants, other than those interviewed in their official capacity, have been changed to protect anonymity.

8 The term “amchi” (Tib. *am chi*) is the most common Tibetan term to refer to practitioners of Sowa Rigpa, used much like the English “doctor”.

9 At the time that I interviewed her, Amchi Sonam Dolma was head of the MTK Translation Department, responsible for translating the different volumes of the *rGyud bZhi* text into English.

10 The umbrella term “*sems nad*” (“illness of the mind”) is often used by both medical practitioners and lay Tibetans to refer to a range of conditions which include psychological symptoms of different kinds.

11 In addition, in some contemporary Tibetan communities, *rlung* has also taken on some broader meanings in the context of current cultural and political circumstances. For example, researchers have described the use of a *rlung* diagnosis to indicate the difficulties of the contemporary political situation in Chinese-occupied Tibet (see, for example, research by Adams 1998; Janes 1999) and in exile (see work by Prost 2006), as well as the use of *rlung* to denote a “modern”, “Western” lifestyle in exile, full of stress and “pressure” (which, as Prost notes, may be positive or negative) (Prost 2006, pp. 124–125).

12 Generally this refers to the Sowa Rigpa practitioner talking with the patient during the appointment, rather than the more structured specific course of “counselling” with a counsellor or therapist which we might think of in the UK or the USA.

13 The current iteration is the *ICD-10: 2010 version* (WHO 2010).

14 The most recent update of this classification system occurred in 2013, in the form of the DSM-5 (APA 2013).

15 For example, the DSM-5 describes symptoms for “major depressive disorder” including “[d]epressed mood most of the day, nearly every day”, “decrease or increase in appetite”, “psychomotor agitation”, “diminished ability to think or concentrate”, and “recurrent suicidal ideation” (APA 2013, pp. 160–161).

16 Thus we find in the *ICD* system, for example, “generalized anxiety disorder” listed under “neurotic, stress-related and somatoform disorders (F40–F48)”, but many disorders including psychosis categorised under “schizophrenia, schizotypal and delusional disorders (F20–F29)” (http://apps.who.int/classifications/icd10/browse/2010/en#/V).

17 Furthermore, other researchers have drawn similarities between *srog rlung* and biomedical categories which are different again—Benedict et al. (2009) for example, have equated of *srog rlung* with post-traumatic stress disorder in exiled Tibetans residing in north India.
